# OxyChip Implantation and Subsequent Electron Paramagnetic Resonance Oximetry in Human Tumors Is Safe and Feasible: First Experience in 24 Patients

**DOI:** 10.3389/fonc.2020.572060

**Published:** 2020-10-27

**Authors:** Philip E. Schaner, Jason R. Pettus, Ann Barry Flood, Benjamin B. Williams, Lesley A. Jarvis, Eunice Y. Chen, David A. Pastel, Rebecca A. Zuurbier, Roberta M. diFlorio-Alexander, Harold M. Swartz, Periannan Kuppusamy

**Affiliations:** ^1^Department of Medicine, Dartmouth Hitchcock Medical Center, Lebanon, NH, United States; ^2^Geisel School of Medicine, Dartmouth College, Hanover, NH, United States; ^3^Norris Cotton Cancer Center, Lebanon, NH, United States; ^4^Department of Pathology, Dartmouth Hitchcock Medical Center, Lebanon, NH, United States; ^5^Department of Radiology, Dartmouth Hitchcock Medical Center, Lebanon, NH, United States; ^6^Department of Surgery, Dartmouth Hitchcock Medical Center, Lebanon, NH, United States

**Keywords:** OxyChip, hypoxia, electron paramagnetic resonance, oximetry, clinical trial, safety, feasibility

## Abstract

**Introduction:** Tumor hypoxia confers both a poor prognosis and increased resistance to oncologic therapies, and therefore, hypoxia modification with reliable oxygen profiling during anticancer treatment is desirable. The OxyChip is an implantable oxygen sensor that can detect tumor oxygen levels using electron paramagnetic resonance (EPR) oximetry. We report initial safety and feasibility outcomes after OxyChip implantation in a first-in-humans clinical trial (NCT02706197, www.clinicaltrials.gov).

**Materials and Methods:** Twenty-four patients were enrolled. Eligible patients had a tumor ≤ 3 cm from the skin surface with planned surgical resection as part of standard-of-care therapy. Most patients had a squamous cell carcinoma of the skin (33%) or a breast malignancy (33%). After an initial cohort of six patients who received surgery alone, eligibility was expanded to patients receiving either chemotherapy or radiotherapy prior to surgical resection. The OxyChip was implanted into the tumor using an 18-G needle; a subset of patients had ultrasound-guided implantation. Electron paramagnetic resonance oximetry was carried out using a custom-built clinical EPR scanner. Patients were evaluated for associated toxicity using the Common Terminology Criteria for Adverse Events (CTCAE); evaluations started immediately after OxyChip placement, occurred during every EPR oximetry measurement, and continued periodically after removal. The OxyChip was removed during standard-of-care surgery, and pathologic analysis of the tissue surrounding the OxyChip was performed.

**Results:** Eighteen patients received surgery alone, while five underwent chemotherapy and one underwent radiotherapy prior to surgery. No unanticipated serious adverse device events occurred. The maximum severity of any adverse event as graded by the CTCAE was 1 (least severe), and all were related to events typically associated with implantation. After surgical resection, 45% of the patients had no histopathologic findings specifically associated with the OxyChip. All tissue pathology was “anticipated” excepting a patient with greater than expected inflammatory findings, which was assessed to be related to the tumor as opposed to the OxyChip.

**Conclusion:** This report of the first-in-humans trial of OxyChip implantation and EPR oximetry demonstrated no significant clinical pathology or unanticipated serious adverse device events. Use of the OxyChip in the clinic was thus safe and feasible.

## Introduction

Tumor hypoxia is associated with a poor prognosis as well as increased resistance to oncologic therapies, including radiotherapy and chemotherapy, in many malignancies (1–5). A multitude of clinical trials have attempted to modify tumor hypoxia in order to improve therapeutic efficacy; for example, a meta-analysis of trials investigating hypoxia modification during radiotherapy in head-and-neck squamous cell carcinomas demonstrated a significant improvement with hypoxia modification in locoregional control [odds ratio (OR) 0.71], disease-specific survival (OR 0.73), and overall survival (OR 0.87) (6). However, routine hypoxia modification in the clinic has generally not been adopted as standard of care. In part, this is due to the failure to demonstrate an overall survival benefit in modern Phase III trials; it has been suggested that this outcome is related to an inability to appropriately select patients for targeted hypoxia interventions (7). Adoption has also been hampered by difficulties associated with the clinical implementation of hypoxia modification in a straightforward, cost-effective manner. Electron paramagnetic resonance (EPR) oximetry has the potential to address these needs by facilitating appropriate patient selection prior to oxygen modification, providing real-time feedback as to the success of oxygen modification, and functioning seamlessly within the clinical workflow.

EPR oximetry has the potential to allow rapid, repeated assessments of hypoxia in the clinical setting (8). EPR oximetry relies on a paramagnetic probe implanted within a tissue of interest to measure the surrounding partial pressure of oxygen (pO_2_). Subsequent oxygen measurements are obtained non-invasively by placing a surface coil of about 10 mm in diameter over the probe. pO_2_ measurements are then obtained in real time as often as desired (8).

EPR oximetry in humans has thus far been conducted using probes composed of ink particulates; these probes are limited in that they can only measure oxygen if placed within a few mm of the skin surface (9, 10). Although ink particulates are tolerated well, this depth limitation hampers their clinical utility *vis a vis* human malignancies (11). In contrast, the OxyChip probe, a small paramagnetic oxygen sensor composed of oxygen-sensing lithium octa-*n*-butoxynaphthalocyanine (LiNc-BuO) crystals embedded in a biocompatible polymer (12), can measure pO_2_ up to a depth of at least 1.5 cm. Here, we report initial data from a first-in-humans trial on the feasibility of OxyChip implantation and EPR oximetry, clinical adverse events associated with OxyChip implantation, and the histopathology associated with the presence of the OxyChip in human tissues. Data pertaining to the primary endpoint of the trial, safety, are reported here. Data pertaining to the secondary endpoint, EPR oximetry measurements, will be reported separately.

## Materials and Methods

**Figure 1:**
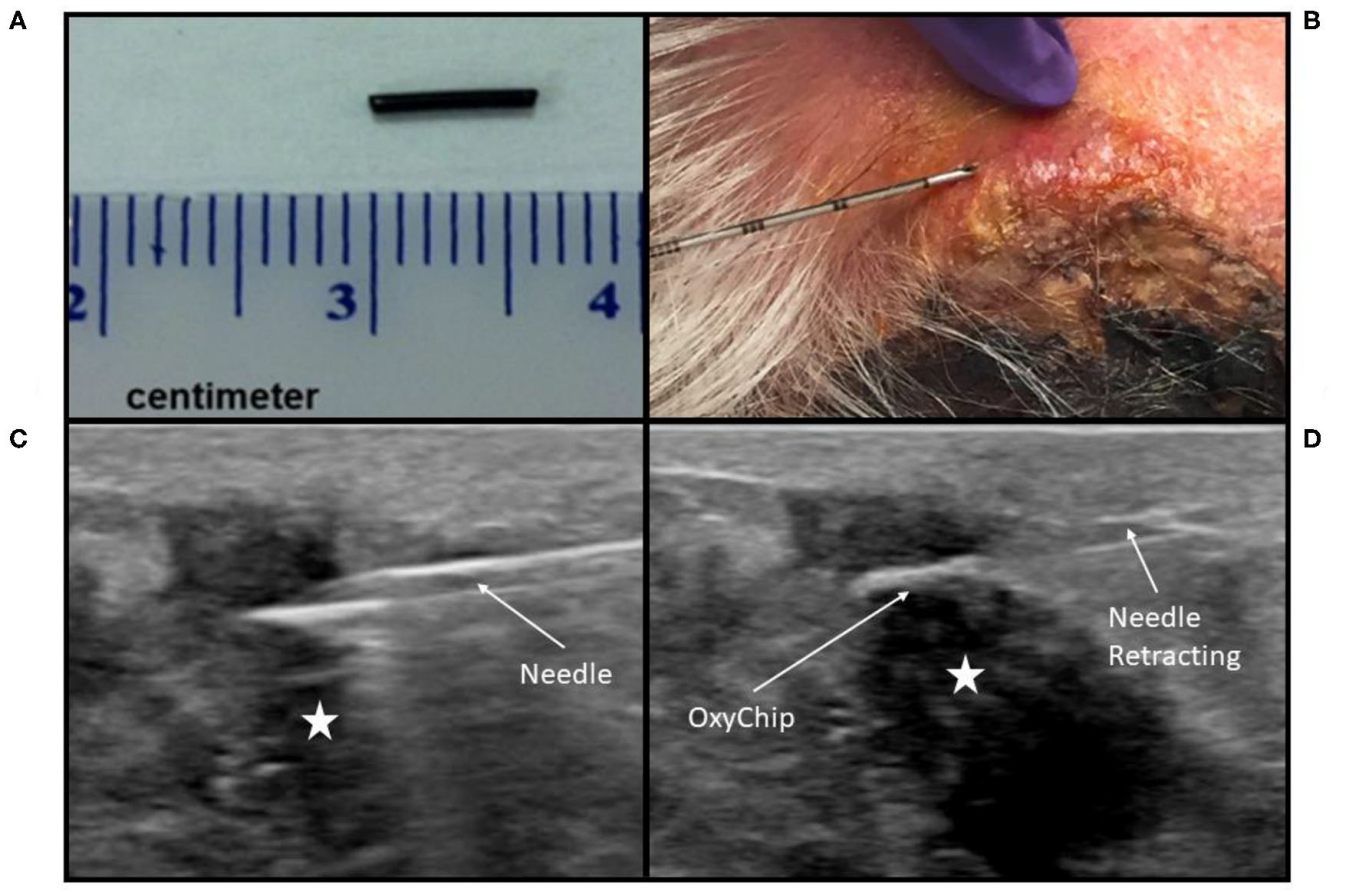
OxyChip implantation. **(A)** OxyChip prior to implantation. **(B)** Implantation needle, with the OxyChip inside, being inserted into a squamous cell carcinoma of the skin. Due to the depth and size of the malignancy, no image guidance was used, and depth of insertion was determined using needle graduations. **(C,D)** Implantation under ultrasound guidance into a breast malignancy. **(C)** The implantation needle, with OxyChip inside, being inserted into the malignancy (hypoechoic area labeled with a star) prior to OxyChip deployment. **(D)** OxyChip after deployment within the malignancy (hypoechoic area labeled with a star). The needle is being retracted after deployment of the OxyChip.

All patients were enrolled in the clinical trial NCT02706197: Oxygen Measurements in Subcutaneous Tumors by EPR Oximetry Using OxyChip. This study was carried out in accordance with US and international standards of Good Clinical Practice (FDA Title 21 part 312 and International Conference on Harmonization guidelines). The Institutional Review Boards (IRBs) at Dartmouth College and Dartmouth-Hitchcock Medical Center approved the protocol (IRB Study 28499). All subjects gave written informed consent in accordance with the Declaration of Helsinki and as approved by these IRBs and the Food and Drug Administration. Eligible patients were 18 years or older, not pregnant, had no contraindications to exposure to a magnetic field, had a tumor ≤ 3 cm from the skin surface (benign tumors, as well as malignancies, were eligible), had not had radiotherapy to the tumor prior to implantation, and were slated to receive surgical resection of their tumor at least 3 days after implantation as part of standard-of-care therapy. An initial cohort of six patients who received surgery alone after OxyChip implantation was evaluated for safety and toxicity endpoints. After this evaluation demonstrated no significant safety or toxicity findings, a second cohort opened, in which patients were allowed to have either chemotherapy or radiotherapy prior to surgical resection, but not both concurrently. The OxyChip, LiNc-BuO crystals embedded in polydimethylsiloxane elastomer, was characterized for human applications according to ISO 10993-12:2012 guidelines (13). Each clinical OxyChip was fabricated in-house to be ~5 mm in length and 0.6 mm in width (Figure 1A) and was sterilized prior to implantation using steam sterilization with appropriate biological and chemical indicators. At the time of implantation, the OxyChip was placed within an 18-G brachytherapy needle, the needle was inserted into the tumor under local anesthesia (1% lidocaine), and the OxyChip was deployed under sterile conditions (Figure 1B). Ultrasound image guidance to direct needle placement was used in a subset of patients (Figures 1C,D). Ultrasound guidance was initially used for deeper tumors or superficial tumors where there was concern on the part of the investigators that deployment might occur outside the tumor. After results indicated OxyChip deployment outside of tumors in a number of superficial sites (see Results), ultrasound guidance was routinely used for most implantations. Implantations occurred either in the clinic or in a dedicated procedural suite depending on the need for image guidance. After implantation, patients were evaluated for associated toxicity by a physician immediately after OxyChip placement, at all EPR oximetry measurements, and, if the patient received chemotherapy, at all chemotherapy administration appointments. Patients were also evaluated within 2 weeks of surgical resection of the tumor and monitored until a year after device removal. Adverse events were scored using the Common Terminology Criteria for Adverse Events (CTCAE) v4.0 (14).

EPR oximetry was carried out using the clinical EPR scanner (8). Patients were positioned supine on a gurney within the magnetic field and the EPR resonator loop was placed over the site of OxyChip implantation. Ultrasound was used in some patients to locate the OxyChip prior to resonator placement. EPR oximetry measurements were performed per protocol with the patient breathing room air, followed by a period of oxygen inhalation using a non-rebreather mask with 100% oxygen at a flow rate of 15 L/min, and then breathing room air again; all three measurement periods were planned for 10 min, for an anticipated total of 30 min. Not all patients completed all measurements, due primarily to logistical or technical considerations (e.g., the patient had a limited time for measurements, or the OxyChip was not found) as opposed to problems tolerating EPR measurements. Measurement sessions were repeated on multiple days as often as the patient was willing and available at the clinic. Patient-reported outcomes were assessed via a questionnaire that was administered to amenable patients (*n* = 4) after the initial EPR oximetry session. Patients were queried using a subjective scale with respect to the implantation as well as the experience of EPR oximetry measurements. The post-measurement questionnaire was considered to be an optional part of the trial, to be added at the discretion of the staff when appropriate. For a variety of reasons, most patients were not invited to complete the questionnaire; for example, in many patients it was felt they did not have enough time to complete the questionnaire due to medical appointments immediately after their EPR oximetry session.

The OxyChip was removed as part of the en-bloc tumor resection during standard-of-care surgery; no OxyChips were removed separately from the main tumor specimen. After surgical removal, gross evaluation of the tissue surrounding the OxyChip was performed. The integrity of the OxyChip, the OxyChip's placement relative to tumor margins, and the distance from the skin surface were assessed. The tissue surrounding the OxyChip was excised and sent for microscopic evaluation; a clinical pathologist assessed tissue findings associated with the OxyChip. The location of the OxyChip relative to microscopic tumor was described and was coded as within the tumor if malignant cells surrounded it; if no malignant cells encompassed the OxyChip, the distance to the closest malignant cell was noted. Adjacent microscopic tissue findings were described, graded in terms of their severity if abnormal, and categorized with respect to whether they were thought related to the implantation procedure and/or the OxyChip itself. They were further categorized as “anticipated” or “un-anticipated” based on expectations of normal tissue response to needle trauma and the presence of a foreign body (15).

## Results

### Patient Population, Implantation Feasibility, and Success Rate

Twenty-four patients were implanted with the OxyChip. The median age was 61 (range 23–83). Forty-six percent were women, and the majority of patients had either a squamous cell carcinoma of the skin (33%) or an invasive ductal carcinoma of the breast (33%) (Table 1). The median time from OxyChip implantation to surgical removal was 29 days (range 4–138). Of the 24 patients implanted, 18 received surgery alone as definitive care for their malignancy (median implant duration 21 days, range 4–42 days); five received chemotherapy prior to surgical resection (median implant duration 131 days, range 124–138 days); and one received radiotherapy prior to surgical resection (implant duration 78 days). In 12 patients, image guidance was not used for placement, initially as the protocol did not incorporate imaging, and later due to the superficiality and size of a number of the malignancies.

**Table 1:** Patient characteristics, clinical adverse events, and pathologic findings associated with OxyChip implantation.

**Patient**	**Age**	**Sex**	**Diagnosis**	**Location of OxyChip implantation**	**US guidance**	**Treatment prior to OxyChip removal**	**Duration of implant (days)**	**Max AE**	**Description AE**	**Location of OxyChip on microscopic evaluation**	**Pathologic findings associated with OxyChip**
1	51	F	Lipoma	Upper left back, subcutaneous	N	None	5	0	NA	Not within tumor; within superficial fascia of subcutaneous mass	Mild macrophage pre-dominant chronic inflammatory reaction at needle site
2	69	F	Melanoma	Left anterior tibia, skin	N	None	4	1	Minor bleeding from implantation needle	Within tumor	Minor hemorrhage at site of injection
3	61	M	SCC Skin	Left nasal dorsum, skin	N	None	32	0	NA	Within tumor	Mild macrophage and foreign body type giant cell reaction at OxyChip site
4	77	M	Melanoma	Scalp, skin	N	None	5	1	Pruritis, scalp	Within tumor	Tumor necrosis and mild hemorrhage immediately adjacent to injection site
5	69	M	BCC	Left temporal scalp, skin	N	None	33	1	Pruritis	Within tumor	Minor focal hemorrhage seen adjacent to the deep margin. Very focal collection of macrophages.
6	63	M	SCC Skin	Scalp, posterior superior, skin	N	None	Unk	0	NA	Not found, presumed lost prior to surgery due to rapidly progressive tumor necrosis	NA
7	61	M	SCC Skin	Right posterior triangle neck, subcutaneous mass	N	None	30	1	Discomfort at surgical site	Outside of and adjacent to tumor within dermis	Focal disrupted tissue at edge of tumor with mild non-specific chronic inflammation
8	56	M	FTC	Thyroid	N	None	47	0	NA	Within tumor	No histologic response seen
9	72	F	SCC Skin	Frontal scalp, left, skin	N	None	7	0	NA	Within tumor	No histologic response seen
10	70	M	SCC Skin	Infraorbital cheek, left, subcutaneous	N	None	25	1	Minor bleeding from implantation needle	Adjacent to tumor, but not within tumor; 0.4 cm from tumor margin	Focal organizing fat necrosis
11	78	M	SCC Skin	Right temporal scalp. Skin	N	None	27	1	Minor bleeding from implantation needle	Within tumor	No histologic response seen
12	83	M	SCC Skin	Right neck, level II lymph node	N	None	22	1	Minor bleeding from implantation needle. Mild bruising.	Within tumor	No comment
13	42	F	IDC	Right breast	Y	None	10	1	Minimal bleeding associated with implantation. Mild bruising at needle insertion site.	Within tumor	No histologic response seen
14	48	F	IDC	Left breast	Y	None	13	1	Minor bleeding from implantation needle. Minor bruising	Not within tumor, 1 mm from tumor edge	Minimal fat necrosis, macrophage infiltrate immediately surrounding the OxyChip
15	70	F	IDC	Left breast	Y	Chemotherapy: paclitaxel/ trasuzumab ×3 cycles	124	1	Mild discomfort from implantation	Uncertain relationship to pre-treatment tumor	No histologic response seen
16	61	F	IDC	Left breast	Y	Chemotherapy: carboplatin/ docetaxel/ trastuzumab/ pertuzumab ×6 cycles	131	1	Mild discomfort and bleeding from implantation procedure. Bruising from implantation needle.	Uncertain relationship to pre-treatment tumor	No histologic response seen
17	61	F	IDC	Left breast	Y	Chemotherapy: dose dense adriamycin/cytoxan ×4 cycles	137	1	Minor bleeding asociated with implantation. Minor bruising near needle insertion site. Mild discomfort of left breast, not specifically associated with area of implantation.	Uncertain relationship to pre-treatment tumor	No histologic response seen
18	23	M	Sarcoma	Right chest wall	Y	Radiotherapy: 50 Gray	78	1	Minor bleeding from implantation needle	Within collagenous soft tissue skeletal muscle fascia outside of viable tumor at least 6 mm	No histologic response seen
19	51	F	IDC	Right breast	Y	Chemotherapy: carboplatin/ docetaxel/ trastuzumab/ pertuzumab ×6 cycles	125	1	Minor bleeding from implantation needle	Uncertain relationship to pre-treatment tumor. OxyChip not seen within small foci of residual tumor.	Focal fibrosis, a few macrophages adjacent
20	55	F	IDC	Left axillary node	Y	Chemotherapy: dose dense adriamycin/ cytoxan ×1 cycle, transitioned to paclitaxel ×1 cycle	138	0	NA	No residual tumor—uncertain relationship to pre-treatment tumor	No histologic response seen
21	81	F	IDC	Right axillary node	Y	None	20	1	Minor bruising associated with implantation site	Freely mobile within necrotic nodal tumor	Focal fibrosis, macrophages, and apparent tumor cavitation
22	65	M	SCC Skin	Above manubrium, skin	Y	None	42	1	Minor bleeding from implantation needle	Within lymph node, adjacent ot nest of tumor	No histologic response seen
23	54	M	SCC HN	Level II LN, neck	Y	None	11	1	Minor bleeding from implantation needle	Within tumor	Tumor necrosis near chip site.
24	53	M	BCC	Face, left, skin	Y	None	Unk	1	Pain and minor bleeding from implantation needle	Not found, presumed lost at time of surgery	NA

*SCC, squamous cell carcinoma; BCC, basal cell carcinoma; FTC, follicular thyroid carcinoma; IDC, invasive ductal carcinoma; AE, adverse events; US, ultrasound*.

Of the patients implanted without image guidance who received surgery alone, at pathological analysis the OxyChip was found within the tumor in 8 of these 11 patients (73%). Three OxyChips were found to be outside but adjacent to the tumor (Table 1, patients 1, 7, 10). In one patient implanted without image guidance, who had a squamous cell carcinoma of the skin, the OxyChip was found neither during EPR oximetry measurement attempts nor on pathologic assessment (Table 1, patient 6). This patient had a rapidly growing and necrotic tumor, which was undergoing daily dressing changes, and it was assumed that the OxyChip was inadvertently dislodged or fell out soon after implantation and prior to initiation of EPR oximetry. Of five patients implanted with image guidance who received surgery alone, four (80%) of the OxyChips were found within the tumor (Table 1, patients 13, 21, 22, 23). In one patient with a basal cell carcinoma who received image-guided placement, the OxyChip was found during EPR oximetry measurements the day prior to surgery but was not found on pathologic assessment after surgical resection (Table 1, patient 24). This patient also had a progressive, necrotic tumor. In this patient, post-surgical MRI of the post-operative bed did not reveal the OxyChip, and it was assumed that the OxyChip had fallen out of the tumor between the last EPR measurement and surgery, or at the time of surgery. Of the five patients treated with neoadjuvant chemotherapy, all OxyChips were within the tumor on initial ultrasound-guided placement (Table 1, patients 15–17, 19, 20); however, determination of the location of the OxyChip relative to the tumor at pathological analysis was confounded by post-treatment effect (i.e., decrease in the size of or complete resolution of the tumor due to a partial or complete response to therapy). In one patient treated with neoadjuvant radiotherapy, the OxyChip was assessed to be within the tumor on initial ultrasound-guided placement (Table 1, patient 18); however, on pathology assessment it was found 6 mm outside the viable tumor. Overall, the OxyChip was definitively found inside the tumor in 50% of patients (*n* = 12, 8 patients without US guidance, 4 patients with US guidance); it was definitively outside the tumor in 21% of patients (*n* = 5, 3 patients without US guidance, 2 patients with US guidance, one of whom received neoadjuvant radiotherapy prior to surgery); its position could not be interpreted due to treatment effect in 21% of patients (*n* = 5, all patients with chemotherapy prior to surgery); it was not found in 8% of the patients (*n* = 2).

### Patient-Reported Outcomes and Adverse Events

No serious unanticipated adverse events occurred. Most clinical adverse events were associated with initial OxyChip implantation. Adverse events noted around implantation were minor bleeding, bruising, and pain. Of these acute events, the maximal severity as graded by CTCAE 4.0 was 1 (i.e., “mild; asymptomatic or mild symptoms; clinical or diagnostic observations only; intervention not indicated”). In no patients were there any overlying skin changes (other than implantation-associated bruising) or signs of infection.

Four patients filled out the post-implantation questionnaire, administered after the first oximetry measurement session (Table 2). Of these, three had surgery alone and one had neoadjuvant chemotherapy. On average, patients rated the pain associated with the initial injection as “a little.” Of the symptoms noted “at any time around the injection site,” two patients rated bleeding as “a little,” two patients noted “a little” tenderness, and two patients noted “a little” pain. For each of these questions, the other two patients responded “not at all.” No patients described swelling, itching, or discharge. With respect to the experience of EPR measurements, all patients rated feeling not at all “closed in, trapped, or unable to get out” of the EPR scanner. Using a 1–10 scale where 10 = “very good” and 1 = “very poor,” the “overall experience” of being measured within the EPR scanner averaged 9, and the “overall comfort” also averaged 9.

**Table 2:** Patient-reported outcomes associated with implantation of the OxyChip and the experience of EPR oximetry.

	**Average score**	**Scale**
**PRO with respect to the OxyChip implant**		
How much pain did you feel the day of the initial injection	3	1: Unbearable; 2: A lot; 3: A little; 4: No pain
At any time did you notice:		1: Not at all; 2: A little; 3: Quite a bit; 4: Very much
Swelling	1	
Itching	1	
Bleeding	1.5	
Tenderness	1.5	
Pain	1.5	
Discharge	1	
**PRO with respect to the EPR oximetry measurement experience**		
Please rate how comfortable you felt:		Scale of 1–5 where 1 is “Very Uncomfortable,” 3 is “Neutral,” 5 is “Very Comfortable”
Lying in the oximetry machine	4	
Being confined in the machine	4.5	
During the measurement, did you ever feel:		1: Not at all; 2: A little; 3: Quite a bit; 4: Very much
Closed in, trapped, or unable to get out	1	
Pain or discomfort	1.75	
How would you rate the time it took to complete the oximetry measurement	1	1: Acceptable; 2: A little too long; 3: Much too long; 4: Unacceptable
How would you rate the following in being measured by oximetry		1–10 scale, where 1 = “Very Poor” and 10 = “Very Good”
Overall experience	9.75	
Overall comfort	9	

### Pathologic Findings Associated With OxyChip Implantation

**Figure 2:**
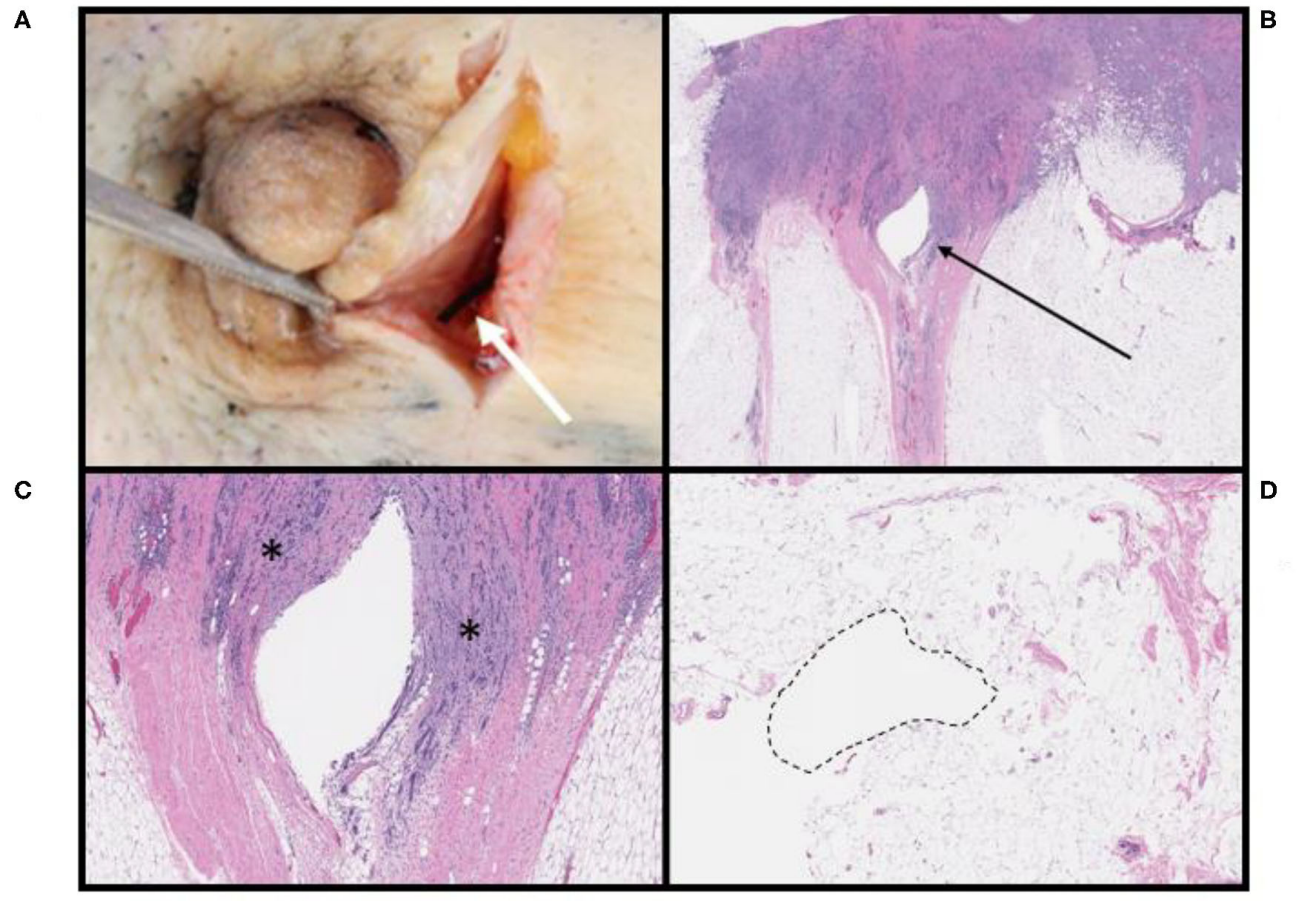
Pathologic findings associated with OxyChip in invasive ductal carcinomas of the breast. **(A)** OxyChip *in situ* (white arrow) adjacent to the nipple within a gross specimen. **(B)** Low-power view of tissue surrounding the OxyChip defect (arrow); the patient received surgery alone 10 days after OxyChip placement. **(C)** High-power view of the same patient in **(B)**. The OxyChip was present within the borders of invasive carcinoma (*). **(D)** Benign adipose tissue surrounding the OxyChip in a patient who received neoadjuvant chemotherapy followed by surgery. The OxyChip was in place for 131 days, and there is no definite evidence of tissue response or inflammation.

**Figure 3:**
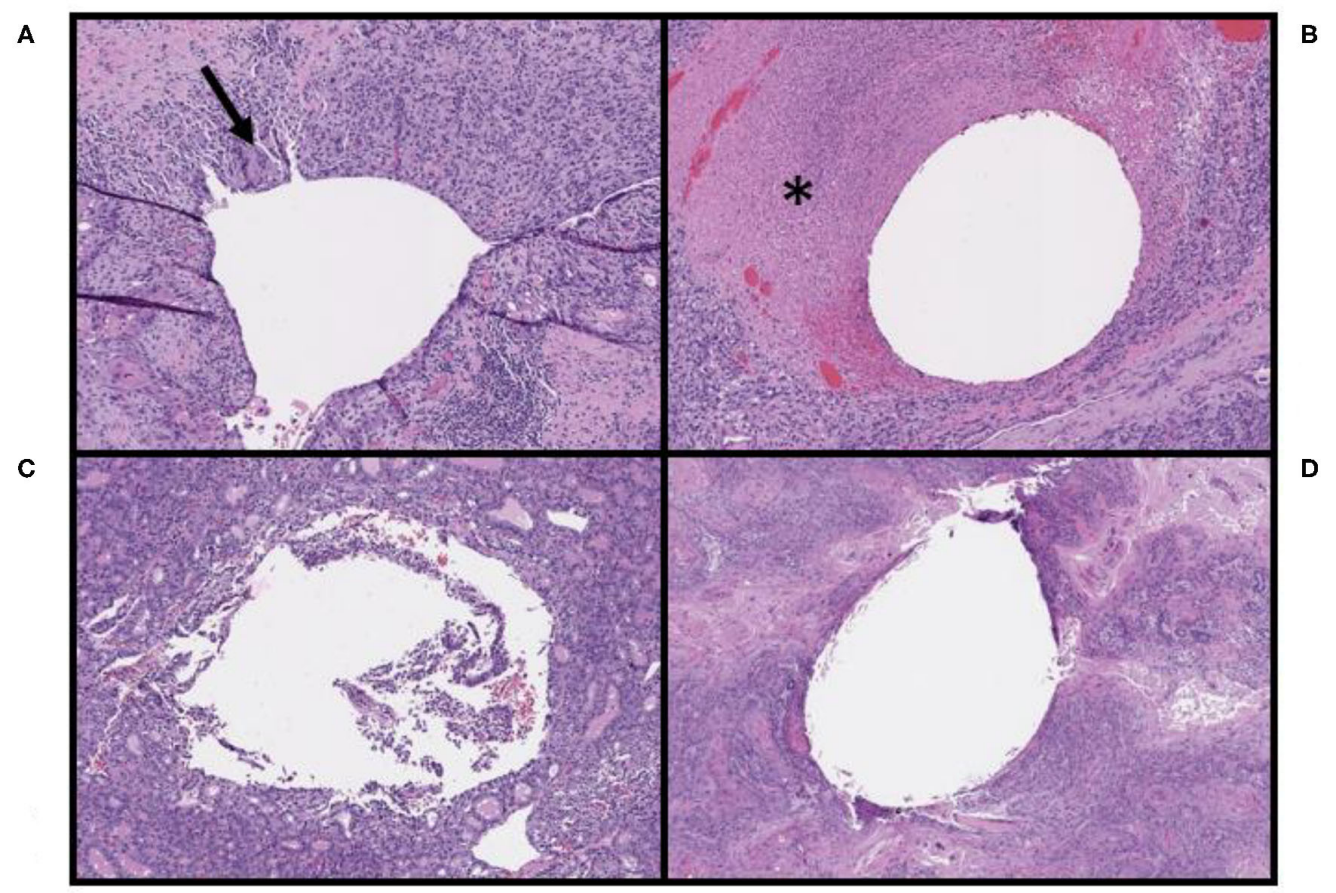
Pathologic findings associated with the OxyChip in non-breast malignancies. **(A)** Tissue surrounding the OxyChip in a patient with a squamous cell carcinoma (SCC) of the left nasal skin, including associated foreign body giant cells (arrow), likely due to tumor keratin reaction at the site of injection. **(B)** Tissue surrounding the OxyChip in a patient with a melanoma of the scalp, including peritumoral injection-related tumor necrosis (^*^). **(C)** Tissue surrounding the OxyChip in a patient with a follicular thyroid cancer, showing no identifiable tissue response. **(D)** Tissue surrounding the OxyChip in a patient with an SCC of the frontal scalp, showing no identifiable tissue response.

After surgical resection of the tumor, microscopic findings demonstrated that 10 of 22 patients (45%) had no histopathologic findings associated with the OxyChip. Expected changes associated with the process of implantation, including mild hemorrhage and minor inflammatory reactions at the needle site and surrounding the OxyChip, were present in 6 of 22 patients (27%), all of whom had the OxyChip removed within ~1 month of injection (range 4–33 days) (Table 1, Figures 2, 3). In two patients where the OxyChip was not in tumor but in adjacent tissue, 0.4 and 0.1 cm from the tumor margin, respectively, focal fat necrosis was noted. In one patient, in whom the OxyChip was within a squamous cell carcinoma of the skin, a foreign body type giant cell reaction was noted. This finding was most consistent with a characteristic keratin foreign-body reaction in a disrupted squamous cell carcinoma. No associated tissue reactions on histopathology were found around the OxyChip in four of the five patients who had neoadjuvant chemotherapy (and only minor focal fibrosis, felt “unlikely” to be related to the OxyChip in the fifth patient), all of whom had the OxyChip implanted for greater than 120 days. Radiation fibrosis was evident in the patient who received prior radiotherapy, but no histologic response attributable to the OxyChip was noted. All pathologic findings were scored as “anticipated” except in a patient with an invasive ductal carcinoma of the breast, in which the OxyChip was implanted into an involved axillary lymph node. The OxyChip in this patient ended up in a cystic core with associated fibrosis and inflammation surrounding this area. As the focal fibrosis/inflammation was deemed more than typical, it was scored as “unanticipated;” however, the histopathologic changes were felt to be a function of the tumor and a heightened immune response secondary to the lymph node site rather than the implantation procedure or the OxyChip itself.

## Discussion

Despite numerous clinical trials investigating hypoxia modification concurrent with anti-neoplastic therapies, hypoxia modification has not yielded a consistent benefit that has translated to routine clinical use. Data strongly indicate that in some circumstances, such as radiotherapy for head and neck cancer, hypoxia modification has the potential to significantly improve oncologic outcomes (6, 16). However, clinical implementation of hypoxia modification even in the most promising circumstances has been hampered by an inability to (i) appropriately identify patients with hypoxic malignancies, (ii) assess whether each individual patient's malignancy will respond to hypoxia modification (and if so, to what type of hypoxia modification), and (iii) reassess the tumoral response to hypoxia modification during treatment (allowing for alteration and adjustment of hypoxia modification during therapy). In short, in the absence of a technique to meet the noted needs, clinical implementation of hypoxia modification has not entered the era of precision medicine. Individualized treatment is likely integral to successful, consistent improvement in oncologic outcomes. Furthermore, these assessments and interventions need to be feasible in the clinic in order to be integrated with current standard-of-care therapies.

The OxyChip has the potential to significantly contribute to the individualization of hypoxia modification therapy in that it can facilitate rapid, repeatable assessment of absolute tumoral pO_2_ in the clinic prior to and during oncologic therapies. In this initial clinical trial, its use was found to be safe and clinically feasible. Of the 24 patients implanted with the OxyChip, no adverse events greater than Grade 1 were noted, and all were associated with the initial minimally invasive implantation procedure. Patient-reported outcomes indicated that the overall process of implantation and measurement was well-tolerated, although the number of patients who agreed to participate in this portion of the trial was small. On microscopic assessment of tissue adjacent to the OxyChip, 45% of specimens had no associated histopathology, and on those specimens that had associated histopathologic changes, all were consistent with the mild inflammation expected with implantation trauma and the presence of a foreign body or tumor-related histopathology. In only one patient was there an “unanticipated” degree of necrosis and inflammation, but these findings were felt to be a function of the tumor and not the implantation procedure or the OxyChip itself. Importantly, four of six long-term implantations (range 78–138 days) had no histopathologic reaction to the OxyChip, and in the other two implantations, only focal, minor fibrosis was present. These data indicate that the process of implantation and measurement was well-tolerated with minimal risk and that tissue reactions to the presence of the OxyChip appeared to be within expectations.

Localization of the OxyChip to the area of interest (i.e., the tumor itself) is critical to the success of targeted hypoxia assessment. It would be clearly desirable to have a high degree of confidence (i) that the OxyChip will be placed within the tumor and (ii) that its location can be easily verified once placement has occurred. In the current trial, in patients who received surgery alone, placement was achieved with an 80% (*n* = 5) to 73% (*n* = 11) success rate, with and without imaging, respectively. It was not possible to definitively assess whether placement was within the tumor in patients who received neoadjuvant chemotherapy or radiation therapy due to treatment response, although at the time of OxyChip deployment it appeared to be within the tumor in all cases. Although ultrasound guidance did not appear to significantly increase the likelihood of intratumoral placement, it is important to note that in general, ultrasound guidance was used for deeper tumors that were not easily assessed on physical exam; the rate of successful placement is likely to have been much lower without ultrasound guidance in these patients. Given clear experiential technical advantages with ultrasound, we continue to use it for assistance with placement in almost all enrolled patients.

In order to maximize the probability of placement within the tumor, patient selection is also important. Although no minimum size criteria was mandated for enrollment earlier in the trial, in order to minimize the risk of placement outside of tumor currently the minimum size of eligible tumors is 2.5 cm. The size of the OxyChip, at 0.5 cm in length, associated with potential uncertainty even with image-guided placement, likely contributed to challenges with deployment into smaller tumors. At this point in the evolution of this technology, however, retrieval of the OxyChip has been necessary in order to appropriately investigate device safety, and smaller sizes were felt to present challenges *vis a vis* this need. Future iterations of the OxyChip may include smaller probes, thereby diminishing the impact of size on accuracy of deployment. Other considerations impact the decision to use differently sized OxyChips, most prominently the issue of hypoxia heterogeneity within a given tumor. Heterogeneity of oxygenation within tumors is a complex issue, as it can vary spatially and temporally and is influenced by underlying pathology as well as anti-neoplastic therapies (17). The OxyChip has unique advantages in terms of assessing temporal changes in hypoxia via EPR oximetry, as the pO_2_ can be repeated measured non-invasively as often as desired, but the measurement is limited to the small volume of tissue surrounding the OxyChip. The use of multiple, smaller OxyChips deployed within different regions of the tumor is an area of investigation, and may provide greater insight into spatially heterogeneous hypoxia.

The clinical relevance of this approach is dependent on successful placement and detection of the OxyChip, and the procedural modifications that have developed over the course of the protocol (e.g., patient selection, use of ultrasound imaging) are expected to increase the likelihood of accurate placement in future trials. Regarding the potential “loss” of OxyChips (i.e., implants not found at the time of pathology), it is difficult to draw conclusions from the two patients in whom the OxyChip was not found due to sample size. However, both patients had superficial, ulcerated skin malignancies, and the OxyChip was placed in viable tumor at the edge of the necrotic area. Based on this experience, it seems reasonable to be cautious about implantations in necrotic, superficial malignancies due to the risk of progressive necrosis and potential loss of the OxyChip. Currently, the OxyChip is not radio-opaque and is also difficult to visualize by ultrasound. It is thus challenging to quickly image the OxyChip prior to or during surgery to confirm its presence and location. In order to mitigate the possibility of OxyChip loss in the future, current efforts are undergoing to modify the OxyChip to increase the sensitivity of routine radiographic and ultrasound imaging.

Current investigational strategies for clinical assessment of tumor hypoxia, and potential hypoxia modification, are beyond the scope of this article and have been reviewed recently (18, 19). Of the strategies that directly measure pO_2_ in tissue, including polarographic electrodes, sensors relying on fluorescence quenching, and EPR oximetry, only EPR oximetry allows repeated measurements in deeper tissues over time in a non-invasive and clinically feasible fashion. The capability to obtain clinically straightforward, repeated measurements over the course of clinical care, interrogating hypoxia both with and without any oxygen modification, is likely to be a necessary component of a successful program of individualized hypoxia modification. The OxyChip device appears to help meet this need, and, as reported here, early data indicate that its use in humans is safe and feasible. Future efforts are focusing on continued assessment of device safety, with the goal of permanent implantation, utilization of pre-implantation hypoxia assessment to facilitate directed OxyChip placement into known areas of hypoxia, and alteration of the number of implantations (and the size of the OxyChip) to increase the yield of information on the spatial heterogeneity of tumor hypoxia. Data from hypoxia measurements in this trial, and the results of hypoxia modification using EPR oximetry with OxyChip, will be reported in a separate publication; it was felt that the extent and complexity of that data would be best served by devoting a single publication to its analysis and that the primary endpoint (safety and efficacy) should be reviewed in depth separately.

## Conclusion

This report of the first-in-humans trial of OxyChip implantation followed by EPR oximetry demonstrated no significant clinical adverse effects. The implantation procedure and the process of EPR oximetry in the clinic were well-tolerated by patients. Histopathologic findings revealed no clinically significant pathology, indicating that the tissue reaction to the OxyChip was well within expectations for an implanted device. Use of the OxyChip in the clinic was thus safe and well-tolerated by patients.

## Data Availability Statement

The raw data supporting the conclusions of this article will be made available by the authors, without undue reservation.

## Ethics Statement

The studies involving human participants were reviewed and approved by The Institutional Review Boards at Dartmouth College and Dartmouth-Hitchcock Medical Center. The patients/participants provided their written informed consent to participate in this study.

## Author Contributions

AF, PS, HS, and PK conceived and designed analysis and wrote the paper. BW, PS, PK, HS, JP, LJ, EC, DP, RZ, and Rd-A contributed data or analysis. PS, AF, and JP performed the analysis. All authors contributed to the article and approved the submitted version.

## Conflict of Interest

AF and HS report part ownership of Clin-EPR, LLC, during the conduct of the study; Clin-EPR manufactures EPR instruments for investigational use only. PK reports that he has a patent issued for OxyChip, but received no compensation. The remaining authors declare that the research was conducted in the absence of any commercial or financial relationships that could be construed as a potential conflict of interest.
